# PHF5A promotes esophageal squamous cell carcinoma progression via stabilizing VEGFA

**DOI:** 10.1186/s13062-023-00440-3

**Published:** 2024-03-01

**Authors:** Zhiwei Chang, Yongxu Jia, Ming Gao, Lijie Song, Weijie Zhang, Ruihua Zhao, Dandan Yu, Xiaolei Liu, Jing Li, Yanru Qin

**Affiliations:** 1 Department of Oncology, The First Affiliated Hospital of Zhengzhou University, Zhengzhou, Henan 450052 P.R. China; 2 State Key Laboratory of Esophageal Cancer Prevention & Treatment, Zhengzhou University, Zhengzhou, Henan 450052 P.R. China

**Keywords:** ESCC, PHF5A, Ubiquitination, VEGFA, PI3K/AKT signaling

## Abstract

**Background:**

Esophageal squamous cell carcinoma (ESCC) is the main subtype of esophageal cancer. Current therapeutic effect is far from satisfaction. Hence, identifying susceptible genes and potential targets is necessary for therapy of ESCC patients.

**Methods:**

Plant homeodomain (PHD)-finger domain protein 5 A (PHF5A) expression in ESCC tissues was examined by immunohistochemistry. RNA interference was used for in vitro loss-of-function experiments. In vivo assay was performed using xenograft mice model by subcutaneous injection. Besides, microarray assay and co-immunoprecipitation experiments were used to study the potential downstream molecules of PHF5A in ESCC. The molecular mechanism between PHF5A and vascular endothelial growth factor A (VEGFA) was explored by a series of ubiquitination related assays.

**Results:**

We found that PHF5A was highly expressed in ESCC tissues compared to normal tissues and that was correlated with poor prognosis of ESCC. Loss-of-function experiments revealed that PHF5A silence remarkably inhibited cell proliferation, migration, and induced apoptosis as well as cell cycle arrest. Consistently, in vivo assay demonstrated that PHF5A deficiency was able to attenuate tumor growth. Furthermore, molecular studies showed that PHF5A silencing promoted VEGFA ubiquitination by interacting with MDM2, thereby regulating VEGFA protein expression. Subsequently, in rescue experiments, our data suggested that ESCC cell viability and migration promoted by PHF5A were dependent on intact VEGFA. Finally, PI3K/AKT signaling rescue was able to alleviate shPHF5A-mediated cell apoptosis and cell cycle arrest.

**Conclusion:**

PHF5A is a tumor promoter in ESCC, which is dependent on VEGFA and PI3K/AKT signaling. PHF5A might serve as a potential therapeutic target for ESCC treatment.

**Supplementary Information:**

The online version contains supplementary material available at 10.1186/s13062-023-00440-3.

## Background

Esophageal cancer is an aggressive and common gastrointestinal cancer type with high lethality [1]. Since low early detection rate and lack of precision therapy, most patients with esophageal cancer showed a poor survival rate [2, 3]. Esophageal squamous cell carcinoma (ESCC) and esophageal adenocarcinoma (EAC) are two major subtypes of esophageal cancer, and the ESCC is more common worldwide than EAC [4]. In spite of the development of multimodality therapies, including surgery combined with chemotherapy and/or radiotherapy, the prognosis of esophagus cancer patients remains poor [5]. Recently, some molecular targets such as EGFR and PI3K/mTOR were used for designing the therapeutic agents including small molecular inhibitor and monoclonal antibody [6–8]. However, no molecular targeting agents contributed to the improvement of survival rate in Phase 3 trials so far [9]. Hence, discovery of new molecular therapeutic targets is important for ESCC treatment.

Plant homeodomain (PHD)-finger domain protein 5 A (PHF5A) is a protein coding gene, belongs to superfamily of PHD-finger and containing a PHD zinc finger domain [10]. PHF5A protein is widely expressed in a variety of eukaryotes from yeasts to human and is highly conserved evolutionarily. It is reported that PHF5A functions as a small transcription factor to elevate its expression by binding to promoter of *connexin43* gene under estrogen stimulus [11]. Besides, as a key member of the splicing factor SF3b complex [12], PHF5A also involved in regulation of downstream genes mediated by RNA splicing pathway [13, 14]. Recent studies have reported that PHF5A not only contributed to the chromatin remodeling [10, 15], tissues and organs morphological development [15], and stem cell pluripotency [16, 17], but also the regulation of cell cycle [18], cell growth and differentiation [10, 17, 19].

Previous investigations have revealed that PHF5A promotes colorectal cancer [20], endometrial adenocarcinoma [21], gastric cancer [22] and glioblastoma stem cell [19] proliferation. However, the role of PHF5A in ESCC development still remain unknown. In this work, we found that PHF5A was highly expressed in esophageal cancer tissues compared to para-carcinoma tissues and that was associated with poor prognosis. Subsequently, PHF5A knockdown suppressed the esophageal cancer cell growth and tumor formation. Further molecular studies demonstrated that the protein stability of VEGFA was regulated by PHF5A, and it was mediated via MDM2 E3 ubiquitin-protein ligase. In-depth rescue experiments illuminated that the promoting role of PHF5A on tumor progression was dependent on the intact VEGFA expression. Furthermore, repaired AKT activation was capable of recovering the PHF5A knockdown-induced disruption in ESCC cells. Collectively, our study indicated that PHF5A played vital roles in ESCC progression and might become a potential therapeutic target for esophageal cancer treatment.

## Results

### High expression of PHF5A was associated with poor progression of Esophageal cancer

**Fig. 1:**
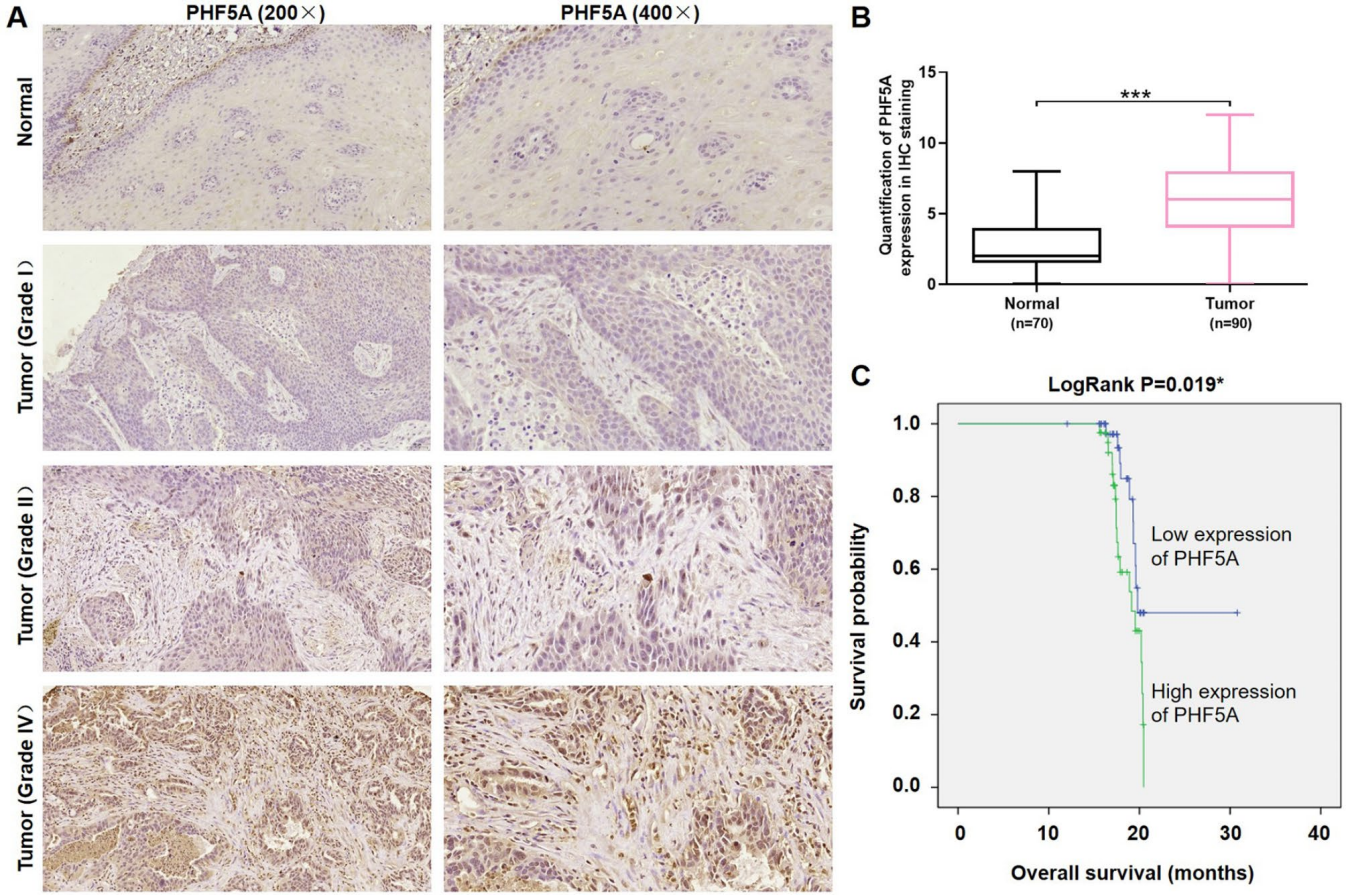
**High expression of PHF5A was associated with poor progression of esophageal cancer.** (**A**) PHF5A expression in normal tissues and esophageal tumor tissues with different tumor grades was determined by immunohistochemistry (Magnification 200× and 400×). (**B**) The quantitative results of PHF5A protein expression in IHC staining. (**C**) Kaplan-Meier survival analysis displayed the relationship between PHF5A expression and patients’ survival period. ***p < 0.001

To study the relationship between PHF5A and ESCC, tissue microarray was applied to detect the characteristics of PHF5A in ESCC tissues with different grades and para-carcinoma normal tissues by immunohistochemistry analysis. As shown by Fig. 1A and B, the expression level of PHF5A in esophageal cancer tissues was significantly elevated in comparison with normal esophageal tissues (*p* < 0.001). Statistical analysis further confirmed that PHF5A high expression is apparently correlated with ESCC tissues other than normal tissues (p < 0.001) **(Table **1). By analyzing the relationship between tumor characteristics and PHF5A expression, we found that tumor infiltration was positively correlated with PHF5A high expression (p < 0.05) **(Tables **2 and 3). In addition, Kaplan-Meier survival analysis was performed and the result demonstrated that high expression level of PHF5A was significantly associated with low overall survival of ESCC patients (*p* < 0.05) **(**Fig. 1C**)**. These results indicated that PHF5A high expression was significantly associated with development and progression of ESCC and might serve as a possible therapeutic target for ESCC treatment.

### PHF5A depletion inhibited cell growth and migration with apoptotic induction and cell cycle arrest

**Fig. 2:**
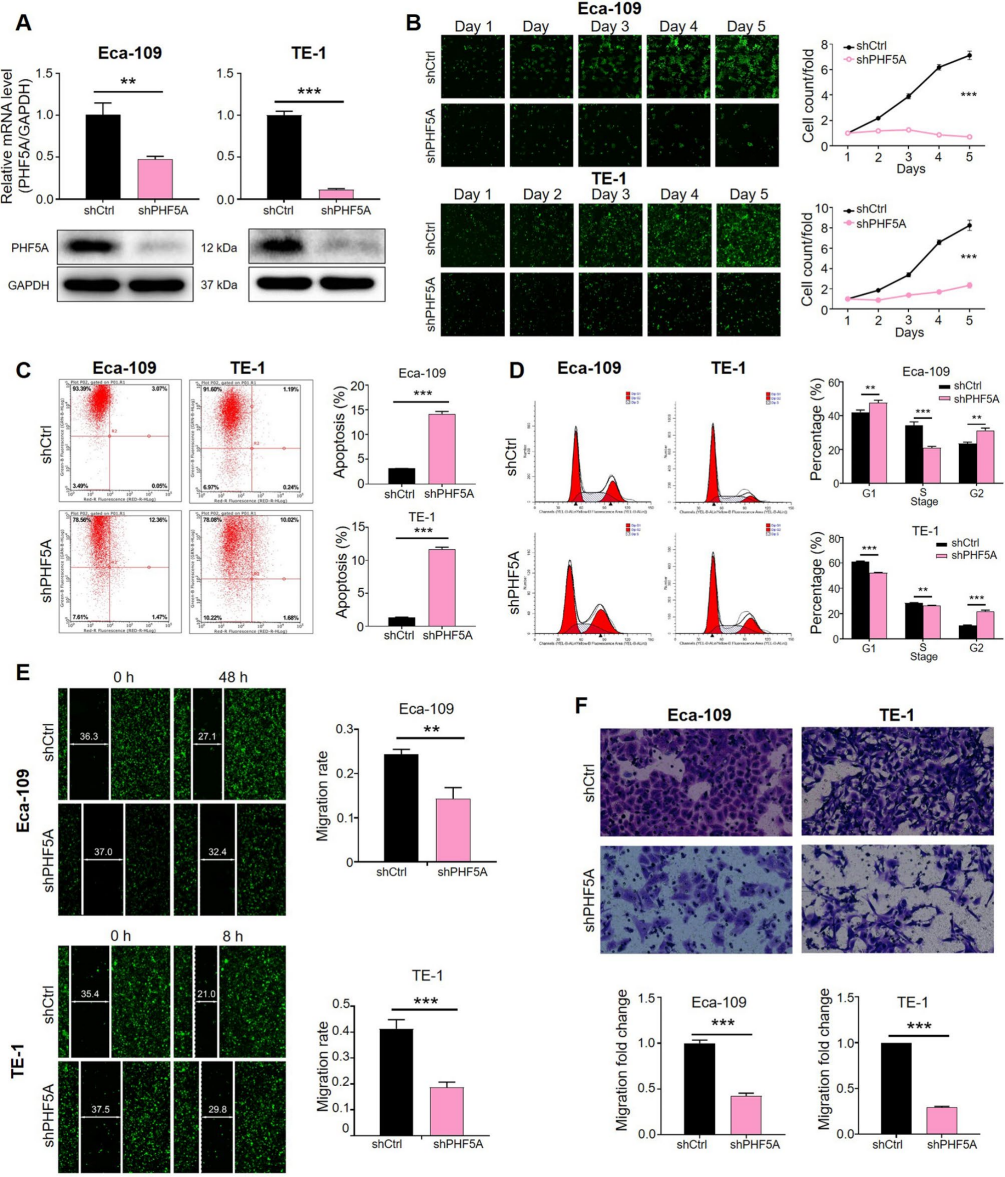
**PHF5A depletion inhibited cell growth and migration with apoptotic induction and cell cycle arrest.** (**A**) PHF5A knockdown efficiency was evaluated at mRNA and protein levels by qPCR and (**B**) western blotting, respectively. GAPDH was used as the internal reference. (**C**) Celigo cell count assay was used for detecting the cell proliferation within five successive days. (**D**) Esophageal cancer cell apoptosis and (**E**) cell cycle distribution was analyzed by flow cytometer after PHF5A silence. (**F**) Wound-healing assay and (**G**) transwell assay were performed to determine cell migration upon PHF5A knockdown. The representative images were randomly selected from at least 3 independent experiments. **p < 0.01, ***p < 0.001

To carry out the loss-of-function experiments, PHF5A downregulated Eca-109 and TE-1 esophageal cancer cell lines were established and the knockdown efficiencies in both cell lines were determined by qPCR and western blotting assays respectively. Both results showed the silencing efficacy is all qualified at mRNA and protein levels (*p* < 0.01) **(**Fig. 2A and B**)**. ESCC cell proliferation upon PHF5A depletion was evaluated by Celigo cell count assay, which displayed a marked suppression in shPHF5A group (*p* < 0.001) **(**Fig. 2C**)**. Furthermore, apoptotic rate of ESCC cells was ascended after PHF5A knockdown, which might be the reason of declined cell viability (*p* < 0.001) **(**Fig. 2D**)**. In addition, we also evaluated the influence of PHF5A on cell cycle distribution, the data suggested that silencing PHF5A could decrease the cell population in S phase and increase the cell percentage in the G2 phase (*p* < 0.01) **(**Fig. 2E**)**. Moreover, wound-healing and transwell assays were performed to unveil alteration of ESCC cell migration upon PHF5A deficiency, our results displayed a similarly significant inhibitions of PHF5A knockdown in migratory ability of ESCC (*p *< 0.01) **(**Fig. 2F and G**)**. Collectively, above results indicated that PHF5A knockdown could inhibit ESCC cell proliferation and migration while inducing apoptosis and cell cycle arrest.

### PHF5A was essential for *in vivo t*umor formation

**Fig. 3:**
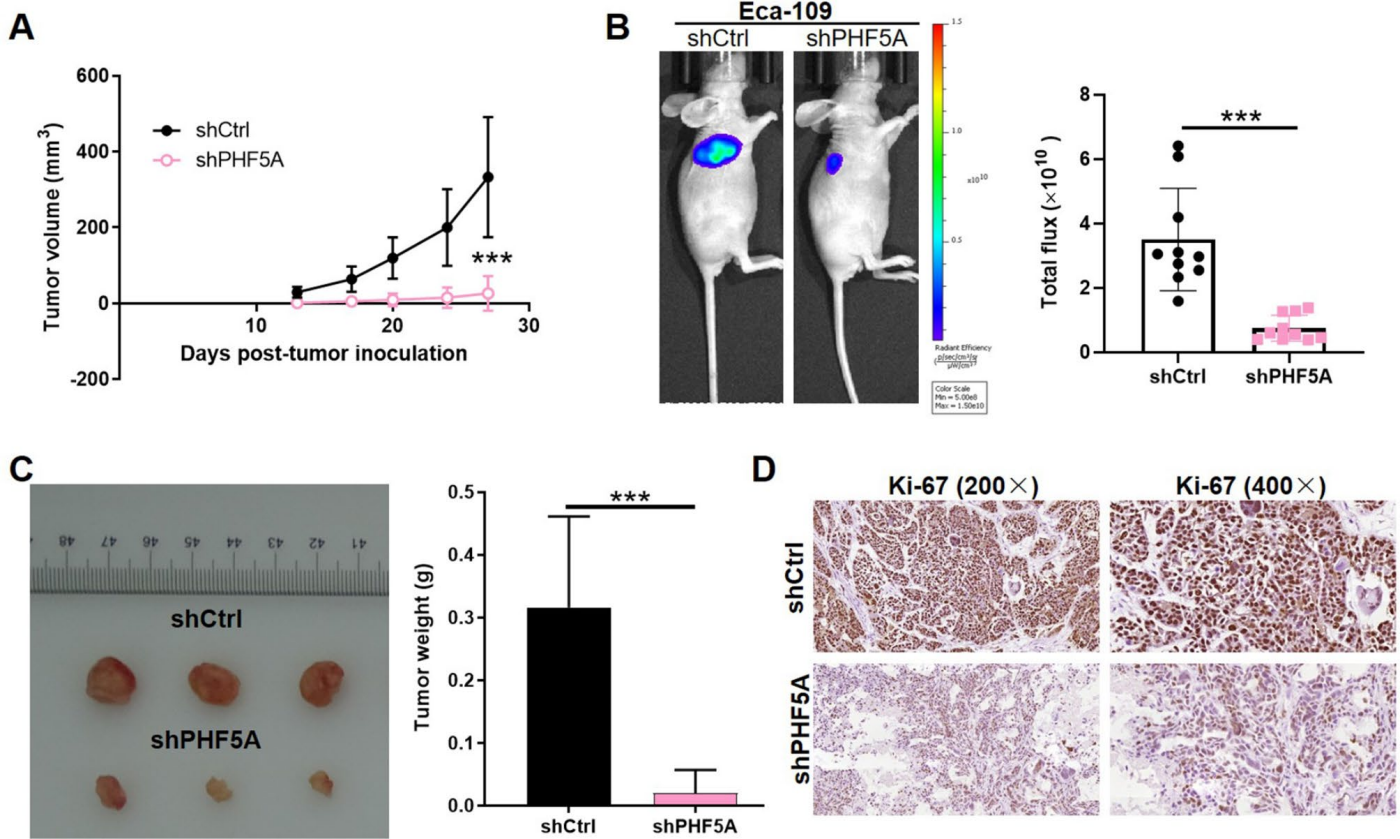
**PHF5A was essential for** in vivo **tumor formation.** (**A**) Tumor volume was calculated based on tumor size measured at indicated time intervals from 13 to 27 days post injection. (**B**) In vivo imaging was performed to evaluate tumor formation. (**C**) After sacrificing mice, xenograft was removed, weighted and photographed for tumor size and weight. (**D**) Immunostaining of Ki-67 was used to analyze the proliferative ability influenced by PHF5A (Magnification 200× and 400×). ***p < 0.001

In order to study the PHF5A roles in tumor formation in vivo, xenograft mice model was employed for observing and recording the tumor growth. 13 days after subcutaneous injection of shCtrl or shPHF5A-infected Eca-109 cells, tumor volume was recorded and calculated by measurements of tumor length and width, showing a remarkable reduction by PHF5A deficit (p < 0.001) **(**Fig. 3A**)**. The in vivo imaging simultaneously displayed smaller tumors in shPHF5A group compared to normal control group (p < 0.001) **(**Fig. 3B**)**. Similarly, after sacrificing the xenotransplant mice, xenografts were removed for recording weight as well as taking photograph. Then, tumor weight was measured as well as tumor size, indicating significant decline by PHF5A knockdown (p < 0.001) **(**Fig. 3C**)**. To analyze the cell proliferation, we further determined the expression of Ki-67, a marker of proliferation, which was remarkably descended in shPHF5A group’s tissue slides using IHC staining **(**Fig. 3D**)**. Taken together, PHF5A was vital for tumor growth in ESCC.

### PHF5A stabilized VEGFA protein via interacting with MDM2

**Fig. 4:**
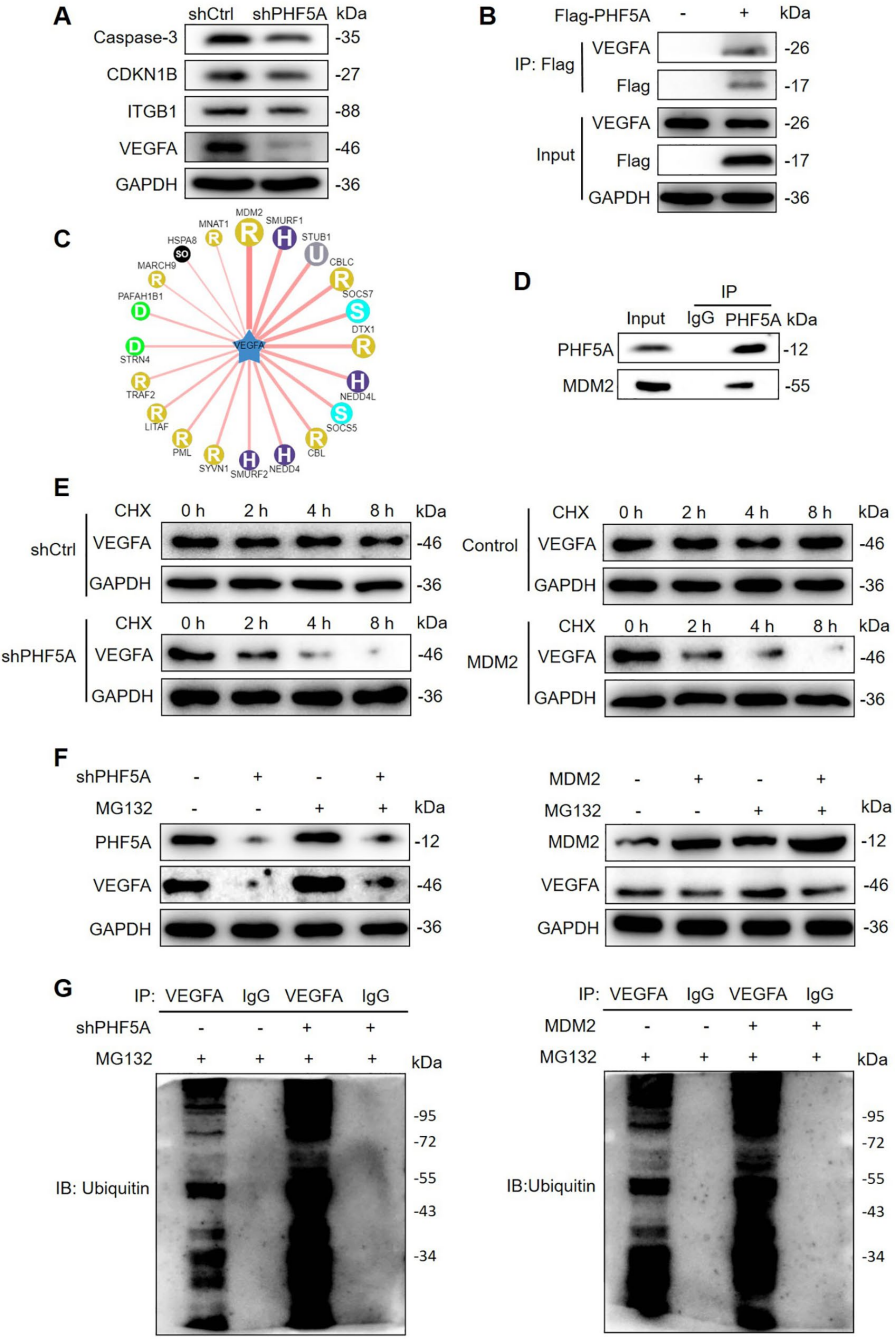
**PHF5A stabilized VEGFA protein via interacting with MDM2.** (**A**) The protein level of potential PHF5A targets were detected by WB analysis. (**B**) The interaction between PHF5A (Flag) and VEGFA was assessed by Co-IP analysis. (**C**) The E3 ligase of VEGFA was predicted using ubibrowser website. (**D**) The endogenous interaction between PHF5A and MDM2 was revealed via Co-IP experiment. (**E**) The VEGFA protein half-life was decreased upon PHF5A depletion or MDM2 overexpression. The cells were treated with 50 µg /mL CHX at the indicated time. (**F**) The addition of MG132 attenuated the VEGFA protein degradation induced by PHF5A depletion or MDM2 overexpression. The transfected Eca-109 cells were treated with 10 µM MG132 for 24 h. (**G**) The endogenous VEGFA protein ubiquitination was promoted by PHF5A silencing and MDM2 overexpressing. The transfected Eca-109 cells were treated with 20 µM MG132 for 6 h. GAPDH served as an internal control

To deeply understand the underlying mechanisms of PHF5A in ESCC progression, the microarray analysis was performed to identify the potential genes regulated by PHF5A depletion. A total of 555 upregulated and 684 downregulated genes were discovered based on the criteria: fold change > 1.3 and FDR < 0.05 **(Fig. ****S1****A)**. We further conducted the pathway enrichment analysis based on IPA software and these differentially expressed genes (DEGs), the data showed that PHF5A depletion could affect several oncogenic pathways, including the mTOR signaling, PI3K/AKT signaling, NF-κB signaling, ERK/MAPK signaling through a key intermediate molecule, the E3 ubiquitin ligase MDM2 **(Fig. ****S1****B)**. Interestingly, we noticed that PHF5A knockdown apparently downregulated the protein expression of VEGFA, while it had no significant effect on the mRNA level of VEGFA **(**Fig. 4A and S1C**)**. Moreover, the results of Co-IP assay suggested that VEGFA directly interacted with PHF5A **(**Fig. 4B**)**.

Considering that the key intermediate molecule MDM2 is an ubiquitin ligase, we hypothesized that PHF5A might regulate VEGFA expression through MDM2-mediated ubiquitination, thus contributing to the ESCC development. Using ubibrowser website (http://ubibrowser.bio-it.cn), we found that MDM2 is an E3 ligase of VEGFA **(**Fig. 4C**)**, and the Co-IP assay showed that PHF5A could bind to MDM2 **(**Fig. 4D**)**. Therefore, we further determined whether the protein stability of VEGFA was affected by PHF5A. The PHF5A-depleted and MDM2-overexpressed Eca-109 cells following CHX treatment were used for observing the half-life of VEGFA protein. Our findings indicated that both silencing PHF5A and overexpressing MDM2 shortened the half-life of VEGFA protein **(**Fig. 4E**)**, while it was reversed by addition of MG132, an inhibitor of proteasome **(**Fig. 4F**)**. These results indicated that PHF5A regulated protein level of VEGFA through ubiquitin proteasome system (UPS)-mediated ubiquitination modification. Subsequent ubiquitination assay demonstrated that the level of VEGFA ubiquitination was enhanced by knocking down PHF5A and overexpressing MDM2 (Fig. 4G). Collectively, PHF5A stabilized VEGFA protein through blocking its ubiquitination.

### ESCC cell progression promoted by PHF5A was dependent on VEGFA

**Fig. 5:**
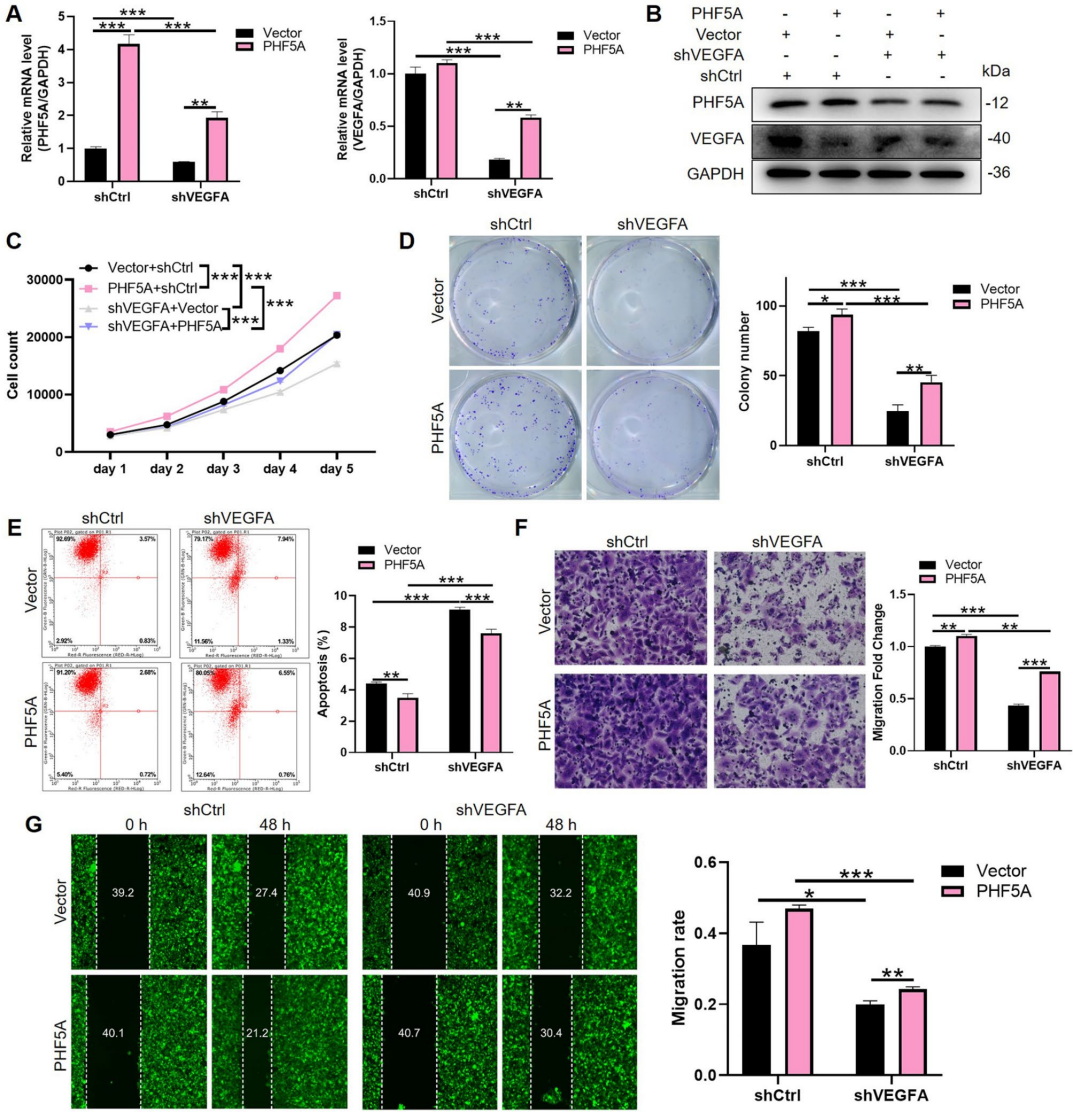
**ESCC cell progression promoted by PHF5A was dependent on VEGFA.** (**A**) Relative mRNA and (**B**) protein levels of PHF5A and VEGFA in Eca-109 cells overexpressed PHF5A, VEGFA knockdown, or simultaneous PHF5A overexpression and VEGFA knockdown were detected by qPCR and WB assays respectively. GAPDH was used as the internal control. (**C**) Celigo cell count assay and (**D**) colony formation assays were used to evaluate growth of Eca-109 cells. (**E**) The roles of PHF5A/VEGFA on Eca-109 cell apoptosis were assessed by flow cytometry. (**F**) Transwell and (**G**) wound-healing assays of PHF5A overexpressing/VEGFA silencing were performed to reveal their regulatory effects on Eca-109 cell migration. The experiments were repeated three times independently. *p < 0.05, **p < 0.01, ***p < 0.001

To analyze the synergistic roles of PHF5A and VEGFA proteins in ESCC cell progression, we firstly overexpressed PHF5A to reconfirm the tumor promotion of PHF5A in ESCC. Moreover, knockdown of VEGFA was applied to study its roles in ESCC growth thereby exploring its synergistic functions with PHF5A. After verification of overexpression or knockdown efficacy **(**Fig. 5A and B**)**, we found that PHF5A overexpression was able to facilitate ESCC cell proliferation while VEGFA silence played an inhibitory role in proliferative capability of ESCC **(**Fig. 5C**)**. Combined both adjustments, the overall results showed that PHF5A was dependent on intact VEGFA for promoting ESCC proliferation. Colony formation assay similarly suggested that proliferative promotion induced by PHF5A required intact VEGFA expression **(**Fig. 5D**)**. In the same way, the level of cell apoptosis indicated that PHF5A was able to alleviate the apoptotic level, whereas VEGFA knockdown aggravate cell apoptosis. Strikingly, the amelioration of apoptosis by PHF5A were compromised due to lack of VEGFA expression **(**Fig. 5E**)**. Both transwell and wound-healing assays showed that PHF5A-mediated migratory promotion of ESCC significantly demanded the robust support from VEGFA **(**Fig. 5F and G**)**. Taken together, above rescue experiments indicated that the synergistic effects of PHF5A and VEGFA on the ESCC cell growth and suggested that VEGFA served as an essential downstream factor of PHF5A contributing to ESCC progression.

### PI3K/AKT signaling was required for PHF5A regulation on ESCC cells

**Fig. 6:**
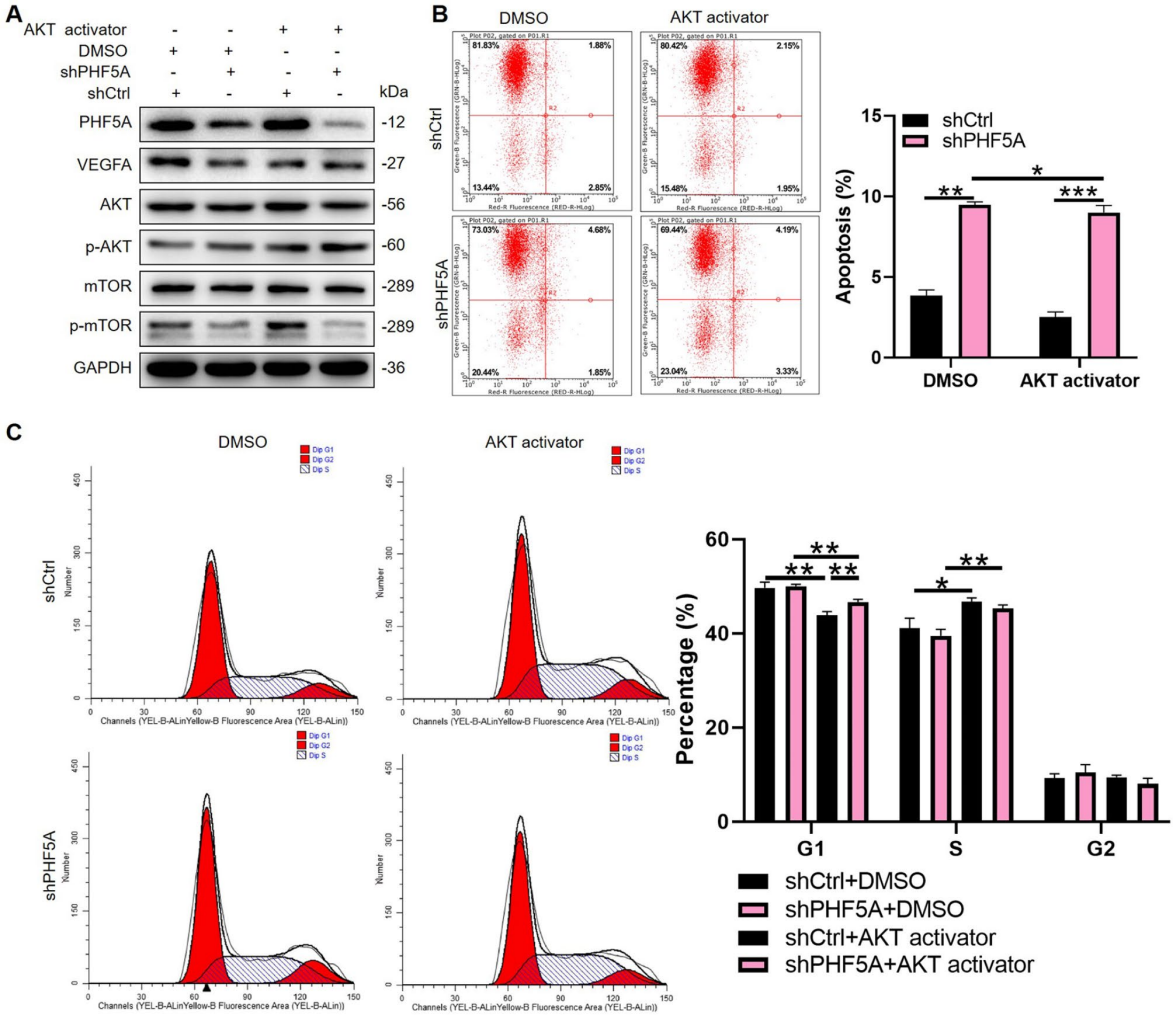
**PI3K/AKT signaling is required for PHF5A regulation on ESCC cells.** (**A**) The PI3K/AKT signaling pathway was inhibited with PHF5A knockdown in Eca-109 cells and this effect was attenuated by AKT activator (SC79, 5 µM). GAPDH was used as the loading control. (**B**) The synergistic effects of PHF5A and AKT activator on Eca-109 cell apoptosis and (**C**) cell cycle was evaluated by flow cytometer. *p < 0.05, **p < 0.01, ***p < 0.001

It is well known that PI3K/AKT/mTOR signaling pathway plays a crucial role in cancer cell proliferation, survival, metastasis and angiogenesis [23], and AKT and mTOR are both downstream targets of VEGFA [24]. Moreover, based on our previous molecular network with different signaling obtained by IPA, we have suggested the possible involvement of PI3K/AKT signaling in PHF5A-mediated ESCC. To deeply figure out the role of PI3K/AKT signaling in ESCC, we further investigated regulation of PHF5A in ESCC with PI3K/AKT signaling replenishment. Thus, PHF5A silence and AKT activator SC79 were applied for examining their role in regulating Eca-109 cell apoptosis and cell cycle. Firstly, the WB data indicated that the phosphorylation of mTOR, a component of the PI3K/AKT pathway was significantly reduced upon PHF5A depletion, and the addition of SC79 activated AKT protein **(**Fig. 6A**)**. Additionally, by analysis of human apoptosis antibody array in Eca-109, we demonstrated that apoptosis related proteins expression, including Bcl-2, CD40, sTNF-1 and XIAP, were decreased in shPHF5A group, and TRAILR-1 protein level was increased with PHF5A deficits **(Fig. S2A and S2B)**. We simultaneously detected PI3K/AKT pathway related proteins, the results showed that the PIK3CA, p-AKT, CDK1 and CCND1 proteins were all downregulated in Eca-109 cells after PHF5A silence, suggesting the inhibitory role of PHF5A knockdown on PI3K/AKT pathway **(Fig. S2C)**. Consistently, as shown in the apoptotic analysis, PI3K/AKT signaling supplement played a vital role in alleviating cell apoptosis induced by PHF5A knockdown **(**Fig. 6B**)**. Similarly, reactivated AKT reverted the cell cycle arrest induced by PHF5A depletion **(**Fig. 6C**)**. Altogether, the PI3K/AKT signaling pathway served as the possible downstream of PHF5A in ESCC development.

## Discussion

As one of the most fatal and aggressive malignancies, patients with esophageal cancer are frequently advanced at the time of detection, thereby contributing to higher mortality [25]. In an effort to ameliorate the outcome of patients after surgery, such patients are often subjected to neoadjuvant concurrent chemoradiotherapy (CRT) for inhibiting tumor growth [26, 27]. However, CRT may induce toxicity levels and potentially lead to surgery delay for patients who is insensitive to CRT. Recently, there are several molecules such as EGFR and PI3K/mTOR were regarded as potentially therapeutic targets. However, the clinical trials of these molecular targeting agents were unsatisfactory [28]. Therefore, identifying valid susceptible gene and therapeutic target is able to help improve the treatment as well as the survival rate.

PHF5A is found to be a key splicing factor as the subunit of splicing factor 3b (SF3b) component, which is involved in the formation of U2 small nuclear ribonucleoprotein (U2 snRNP) splicing complex to promote tumor progression [10]. Specifically, PHF5A can promote the interactions between the U2 snRNP complex and ATP-dependent helicases, further regulating the pre-mRNA splicing of corresponding genes [13]^,^ [14]. In breast cancer, PHF5A expression exhibited a high level and correlated with poor survival. Consistently, in our study, we found that PHF5A was highly expressed in esophageal tumor tissues compared to normal esophageal tissues, which was associated with poor survival. Besides, the high expression of PHF5A was significantly associated with tumor infiltration. Furthermore, it is reported that PHF5A promotes breast cancer and lung adenocarcinoma as a novel oncogene through inhibiting apoptosis [29–31]. We here suggested that PHF5A knockdown was capable of suppressing ESCC cell growth, migration in vitro and tumor growth in vivo by performing further loss-of-function experiments. These results indicated the promoting role of PHF5A in esophageal tumor progression.

Mechanism studies indicated that and PHF5A was essential for SF3b spliceosome stability, and the PHF5A-SF3b complex regulated alternative splicing in apoptotic signaling by linking to histones [32, 33]. Additionally, the PHF5A can also influence Fas-mediated cell apoptosis by negatively controlling expression of short FAS-activated serine/threonine kinase (FASTK) [31]. In lung adenocarcinoma, PHF5A functioned as an oncoprotein to inhibit cisplatin-induced apoptosis, facilitate cell proliferation, migration and invasion, as well as G0/G1 cell cycle progression [29]. However, pladienolide, a small molecular inhibitor of PHF5A, inhibited lung adenocarcinoma cell proliferation in a dose-dependent manner and induced alternative splicing changes [29]. It seems that PHF5A was associated with different apoptotic pathway and alternative splicing in several cancer types. Whether PHF5A knockdown influences the alternative splicing for cell apoptosis in ESCC still need to be further elucidated. To deeply explore the underlying mechanism of PHF5A-mediated ESCC progression, we screened several potential downstream molecule by RNA-sequencing in ESCC cells with or without PHF5A knockdown. Our data revealed that VEGFA was a potential downstream molecule, while its protein expression level rather than mRNA level was downregulated upon PHF5A depletion. Simultaneously, we found MDM2 could link PHF5A to several oncogenic pathways, including the PI3K/AKT signaling, and is a key intermediate molecule. MDM2 is function as an oncoprotein, it plays a promoting role in tumor progression by abrogating the antiproliferative activity of p53 [34]. Importantly, MDM2 is a p53-specific E3 ubiquitin ligase that monoubiquitinates p53, thereby mediating its degradation and limiting the p53 growth-suppressive function [35]. However, MDM2 is also involved in ubiquitination of other proteins, such as parkin interacting substrate (PARIS) [36]. Hence, we hypothesized that PHF5A might regulate VEGFA expression through MDM2-mediated ubiquitination, thus contributing to the ESCC development. As expect, our subsequent Co-IP and ubiquitination related analysis demonstrated this hypothesis, and the synergistic function of VEGFA and PHF5A in promoting ESCC development was validated by in vitro rescue experiments. Altogether, PHF5A elevated VEGFA protein expression by inhibiting MDM2-mediated ubiquitination, thereby promoting ESCC development.

Growth of solid tumors is accompanied by stimulation of angiogenesis [37]. Vascular endothelial growth factor A (VEGFA) is one of the primary factors driving expansion of the tumor vascular bed [38]. Recently, several studies reported that VEGFA was associated with esophageal cancer development [39–41]. For examples, apigenin could suppress VEGF expression and tumor-induced angiogenesis thereby inhibiting esophageal carcinogenesis [39]. Dihydroartemisinin elevated the sensitivity of photodynamic therapy via NF-κB/HIF-1α/VEGF pathway in esophageal cancer cell [40]. ANXA2 promoted esophageal cancer progression by activating MYC-HIF-1α-VEGF axis [41]. These findings suggested that VEGF played essential roles in esophageal cancer progression. In this study, we found that PHF5A interacted with VEGFA, and both molecules played essential roles in promoting ESCC cell growth and migration. Recent investigations reported that HIF-1α/VEGF pathway contributed to esophageal cancer development [40, 41]. However, whether PHF5A is involved in this regulatory pathway in esophageal carcinogenesis seems unclear and concrete sequential regulation of these molecules and signal transduction still need more elucidation. Especially, VEGF is associated with angiogenesis that is important for tumor growth in vivo [42]. The underlying mechanisms of PHF5A and VEGFA would require more related studies to unravel the tumor-promoting details. Whether roles of PHF5A played in the angiogenesis may help us to further understand the principal contribution of the tumor growth. Besides, in our study, PHF5A silence could decline the p-mTOR level, and the AKT agonist could counteract the effects of PHF5A depletion on cell apoptosis and cell cycle, indicating that PHF5A was dependent on PI3K/AKT signaling pathway to facilitate the cell growth.

In conclusion, we discovered that PHF5A expression was significantly elevated in human esophageal cancer and that correlated with a poor prognosis. The biological phenotype studies revealed that PHF5A promoted ESCC cells proliferation and metastasis in vitro, as well as the deriving of tumor growth in vivo. Moreover, we demonstrated that PHF5A could deubiquitinate VEGFA protein via interacting with MDM2, which further leads to enhance PI3K/AKT pathway. Therefore, our findings suggested that PHF5A may function as a promising therapeutic target of ESCC.

## Methods

### Tissue microarray

Human tissue chip containing 90 cases esophagus cancer tissues and 70 cases normal esophageal tissues was used for determining differential expression of PHF5A. Patient information and written informed consents were collected. For immunohistochemical (IHC) staining, dewaxed and rehydrated tissue slides were blocked and incubated with primary antibody PHF5A and followed by secondary antibody conjugation. Diaminobenzene and hematoxylin were used for DAB color development, and staining slides were photographed with microscopy and observed by ImageScope and CaseViewer. All slides were analyzed randomly by two independent pathologists and IHC outcomes were determined by staining percentage and intensity ranks. Staining percentage scores were classified as: 1 (1–24%), 2 (25–49%), 3 (50–74%) and 4 (75–100%). Staining intensity were scored 0 (weak color) to 3 (strong brown). The study was approved by the Ethics Committee of scientific research/drug clinical trial of the First Affiliated Hospital of Zhengzhou University. Antibodies used in IHC were listed in Table S1.

### Cell culture, transfection and chemicals

Eca-109 and TE-1 cells were purchased from the Cell Bank of Type Culture Collection of Chinese Academy of Science and were cultured in RPMI-1640 medium (Gibco) with 10% fetal bovine serum (Gibco). All cells were cultured in a humidified culture incubator at 37 °C under 5% CO_2_ with culture medium changed every 72 h. AKT activator SC79 (Beyotime) was applied at the final concentration 5 µM.

To knockdown or overexpress target gene, prepared lentiviral vectors were transfected to Eca-109 or/and TE-1 cells using Lipofectamine 2000 (Thermo) according to manufacturer’s instructions. Transfection efficacy was determined by green fluorescent protein signal at 72 h post-transfection.

### RNA interference and plasmids packaging

The shRNA sequences targeting human PHF5A and VEGFA were designed and cDNA were synthesized and subsequently cloned into luciferase-labeled BR-V-108 vector. Additionally, PHF5A was amplified and cloned into the BR-V112 vector after double digestion followed by sequencing. Lentiviral particles were collected, following co-transfection using pHelper 1.0 and pHelper 2.0 vector for plasmids packaging. The sequences used were listed in Table S2.

### RNA isolation and qRT-PCR

After 72 h for PHF5A and/or VEGFA RNA expressing, Eca-109 and TE-1 cells in triplicate were fully lysed and total RNA was extracted using TRIzol reagent (Thermo). RNA concentration was determined using Nanodrop 2000/2000 C spectrophotometer (Thermo). cDNA was reversely transcribed from RNA using M-MLV Kit (Promega) and qPCR was performed with SYBR Green mastermixs Kit (Vazyme) and Biosystems 7500 Sequence Detection system. Glyceraldehyde 3-phosphate dehydrogenase (GAPDH) was used as inner control, and the primers used for the PCR reaction were shown in Table S3. The relative quantitative data of gene expression was processed by the 2^−ΔΔCt^ method.

### Western blotting (WB), co-immunoprecipitation (Co-IP) and human apoptosis antibody assay

Cells were harvested after transfection and were lysed in ice-cold radioimmunoprecipitation assay buffer (Millipore), and proteins were collected and were quantified by a BCA Protein Assay Kit (HyClone-Pierce). 20 µg protein samples per lane were loaded and separated by 10% sodium dodecyl sulfate polyacrylamide gel electrophoresis (Invitrogen), and transferred onto polyvinylidene difluoride (PVDF) membranes in ice. Then the membranes were blocked with tris-buffered saline tween-20 solution of 5% slim milk at room temperature for 1 h. Next, membranes were incubated with primary antibodies at 4 °C overnight followed by incubation of secondary antibody HRP goat anti-rabbit/mouse IgG for 2 h at room temperature. The blots were visualized by enhanced chemiluminescence (Amersham). The antibodies used in western blot were listed in Table S1.

For Co-IP assay, total proteins were collected and subsequent Co-IP assay was applied for identifying the interaction between proteins. 1.0 mg total proteins were incubated with anti-DYKDDDDK Tag (binds to the same epitope as Sigma’s Anti-FLAG® M2 Antibody) at 4 °C overnight. Twenty microliters agarose beads were added and incubated at 4 °C for 2 h. After centrifugation at 2000×g for 1 min, supernatant was discarded, and protein A/G beads were harvested and washed twice. Next, the protein A/G beads were denatured in IP lysate buffer and 5× loading buffer at 100 °C for 5 min. Finally, 20 µg protein sample was subjected to WB analysis as described above.

For human apoptosis antibody assay (Abcam), related proteins in human apoptosis signaling pathway were detected according to manufacturer’s instructions. After protein samples collected and quantified, antibody arrays were incubated with protein samples (0.5 mg/mL) overnight at 4 °C followed by cocktail of biotin-conjugated antibodies overnight at 4 °C. Next, chips were incubated with labelled streptavidin for 2 h. Enhanced chemiluminescence was used for visualizing and spots gray value was analyzed by Image J.

### Cell proliferation assays

For Celigo cell counting assay, infected cells were cultured for 72 h and then the cells were harvested and seeded into 96-well plates (2000 cells/well). Cells were further cultured in MEM with 10% FBS at 37 °C with 5% CO_2_ for 5 days. MEM medium was changed every 3 days. Celigo image cytometer (Nexcelom Bioscience) was applied for cell counting at 1–5 day and the cell proliferation curve was graphed.

For colony formation assay, infected cells in the logarithmic growth phase were seeded into six-well plates (1000 cells/well) in triplicate and further cultured for 8 days with the culture medium exchanged every 3 days. Cell clones were photographed under a microscope. Next, all clones were fixed by 4% paraformaldehyde, stained by Giemsa, and were photographed with a digital camera. Colony forming rate equals colony number/inoculated cell number×100%.

### Cell apoptosis and cell cycle assay

Lentivirus infected cells were seeded 6-well plates in triplicate and cultured for another 5 days. Then, cells were harvested and washed with 4 °C ice-cold D-Hanks. After centrifugation 1000×g, cells were resuspended with binding buffer, then 5 µL Annexin V-APC (eBioscience) was added for staining in the dark. Apoptosis analysis was measured using FACS Calibur (BD Biosciences).

For cell cycle analysis, cells were prepared and stained by Propidium Iodide solution (Sigma). Cell cycle distribution was detected by FACS Calibur and observed micropublisher (Olympus).

### Cell migration assay

For wound-healing assay, cells were seeded into 96-well plates with a density of 50,000 cells/well in 100 µL medium and cultured at 37 °C with 5% CO_2_. When cells grew more than 90% confluence, a scratch was made using a scratch tester paralleled the center of the lower end of the 96-well plate. The cells were washed with PBS twice and cultured in 0.5% PBS with 5% CO_2_ at 37 °C. Photographs were captured by Cellomics (ArrayScan VT1, Thermo) at the indicated time point (0 h, 8 and 48 h) and analyzed the migration area with Cellomics.

Transwell assay was performed by Transwell Kit (Corning). Infected cells were collected, counted and incubated in the upper chamber with 100 µL medium without FBS in a 24-well plate (6 × 10^4^ cells/well) in triplicate. Six hundred microliters of medium supplemented with 30% FBS was added in the lower chamber. After incubation at 37 °C with 5% CO_2_, nonmetastatic cells were removed with a cotton swab. Four hundred microliters of Giemsa were added for staining. For the assessment of transwell assay, 5 fields of the lower chamber were randomly selected and the stained cells were counted under 200× microscope. The migratory cell number was determined by average cell number of the 5 microscopic views.

### Mice xenograft model

All animal studies were approved by Institutional Animal Care and Use Committee of Zhengzhou University. Female BALB/c nude mice were purchased from Beijing Vital River Laboratory Animal Technology Co., Ltd. For tumorigenicity, 5 × 10^6^ lentivirus (shCtrl or shPHF5A) transfected Eca-109 cells were subcutaneously injected into each mouse (4-week-old, n = 10 per group). Mice’s weight and tumor sizes were recorded and the volume of tumor was calculated as π/6×L×W^2^ (W, width at the widest point; L, perpendicular width). For in vivo imaging assay, the mice were anesthetized with intraperitoneal injection of 0.7% pentobarbital sodium (Sigma,10µL/g) [43], and the tumor burden was assessed by analysis of fluorescence intensity with the small animal multi-spectral living imaging system (Berthold Technologies).

### Ki-67 staining

Mice tumor sections were fixed in 4% paraformaldehyde. Paraffin embedded 5 μm sections were prepared for hematoxylin, eosin and IHC staining. We added citric acid buffer for antigen retrieval at 120 °C. Sections were blocked using PBS-H_2_O_2_ with 0.1% Tween-20. Ki-67 antibody was added for incubating at 4 °C overnight and then secondary antibodies were used as well. DAB color was displayed with diaminobenzene and hematoxylin. Stained slides were recorded with a microscopy.

### Microarray assay

Total RNA from Eca-109 cells with or without PHF5A was extracted using TRIzol. RNA quantity and quality were evaluated with a Nanodrop 2000 (Thermo). RIN value was evaluated with Agilent 2100 and Agilent RNA 6000 Nano Kit. Affymetrix PrimeView Human Gene Expression Arrays and Affymetrix Scanner 3000 (Affymetrix) were utilized for microarray analysis to obtain gene expression profiles according to the manufacturer’s instructions. Differentially expressed genes (DEGs) were selected based on p < 0.05 and fold change more than 1.3 times. Bioinformatic analysis of DEGs based on Ingenuity Pathway Analysis (IPA) (Qiagen) was performed, and |Z-score|>2 is considered meaningful.

### Bioinformatics analysis

The E3 ubiquitin ligases of VEGFA were predicted through the Ubibrowser website (http://ubibrowser.bio-it.cn/ubibrowser/home/index).

### Protein stability assay and ubiquitination assay

To observe protein stability, the Eca-109 cells with PHF5A-depletion or MDM2 overexpression were treated with 50 µg /mL cycloheximide (CHX) and collected at indicated time points. The cell lysate was subjected to immunoblotting. For ubiquitination assays, the proteasome inhibitor MG132 (20 µM) was added after the transfection of shPHF5 or MDM2 and the cells were incubated for 6 h. Subsequently, the VEGFA or IgG antibodies were added to the cell lysate before incubation overnight at 4 °C. The ubiquitin was detected using an ubiquitin antibody. The detailed information about antibodies used here were listed in Table S1.

### Statistical analysis

Data from independent experiments are shown as the mean ± standard deviations (SD). Statistical analysis between two groups was performed by Student’s *t*-test (two-tailed). PHF5A expression difference between ESCC tissues and normal tissues was analyzed with Rank Sum test analysis. The relationship of PHF5A expression and tumor characteristics in ESCC patients were analyzed with Mann-Whitney U analysis and Spearman Rank correlation analysis. Survival data were evaluated by using Kaplan-Meier survival analysis. *P* value < 0.05 was considered as statistically significant difference.

**Table 1:** PHF5A expression patterns in esophagus cancer tissues and para-carcinoma tissues was revealed by immunohistochemistry analysis

PHF5A expression	Tumor tissue		normal tissue		*P* value
	Cases	Percentage	Cases	Percentage	< 0.001
Low	46	51.1%	68	97.1%	
High	44	48.9%	2	2.9%	

**Table 2:** Relationship between PHF5A expression and tumor characteristics in patients with esophagus cancer

Features	No. of patients	PHF5A expression		*P* value
		low	high	
All patients	90	46	44	
Age (years)				0.143
≤ 64	46	27	19	
> 64	44	19	25	
Gender				0.529
Male	72	38	34	
Female	18	8	10	
Lymph node positive				0.403
≤ 1	47	26	21	
> 1	41	19	22	
Tumor size				1.000
≤ 5 cm	46	23	23	
> 5 cm	34	17	17	
Grade				0.155
I	7	4	3	
II	49	22	27	
III	25	17	8	
AJCC Stage				0.074
1	3	3	0	
2	30	19	11	
3	33	12	21	
4	3	3	0	
Tumor Infiltrate				< 0.05
T0	1	1	0	
T1	3	2	1	
T2	15	10	5	
T3	39	21	18	
T4	11	3	8	
Lymphatic metastasis (N)				0.293
N0	30	19	11	
N1	18	7	11	
N2	12	7	5	
N3	9	4	5	

**Table 3:** Pearson correlation analysis between PHF5A expression and tumor characteristics in patients with esophagus cancer

Tumor characteristics	Index	PHF5A
Tumor Infiltrate	Pearson correlation	0.255
	Significance (two-tailed)	< 0.05
	N	69

### Electronic supplementary material

Below is the link to the electronic supplementary material.

Supplementary Material 1

## Data Availability

All data generated or analysed during this study are included in this published article and its supplementary information files.
