# Supplementary material for: PHF5A promotes esophageal squamous cell carcinoma progression via stabilizing VEGFA
Source: Biol Direct. 2024 Mar 1;19:19. doi: 10.1186/s13062-023-00440-3 (PMC10905922; doi:10.1186/s13062-023-00440-3)
Supplement: Supplementary file 1 — Supplementary Material 1 [file 13062_2023_440_MOESM1_ESM.docx]

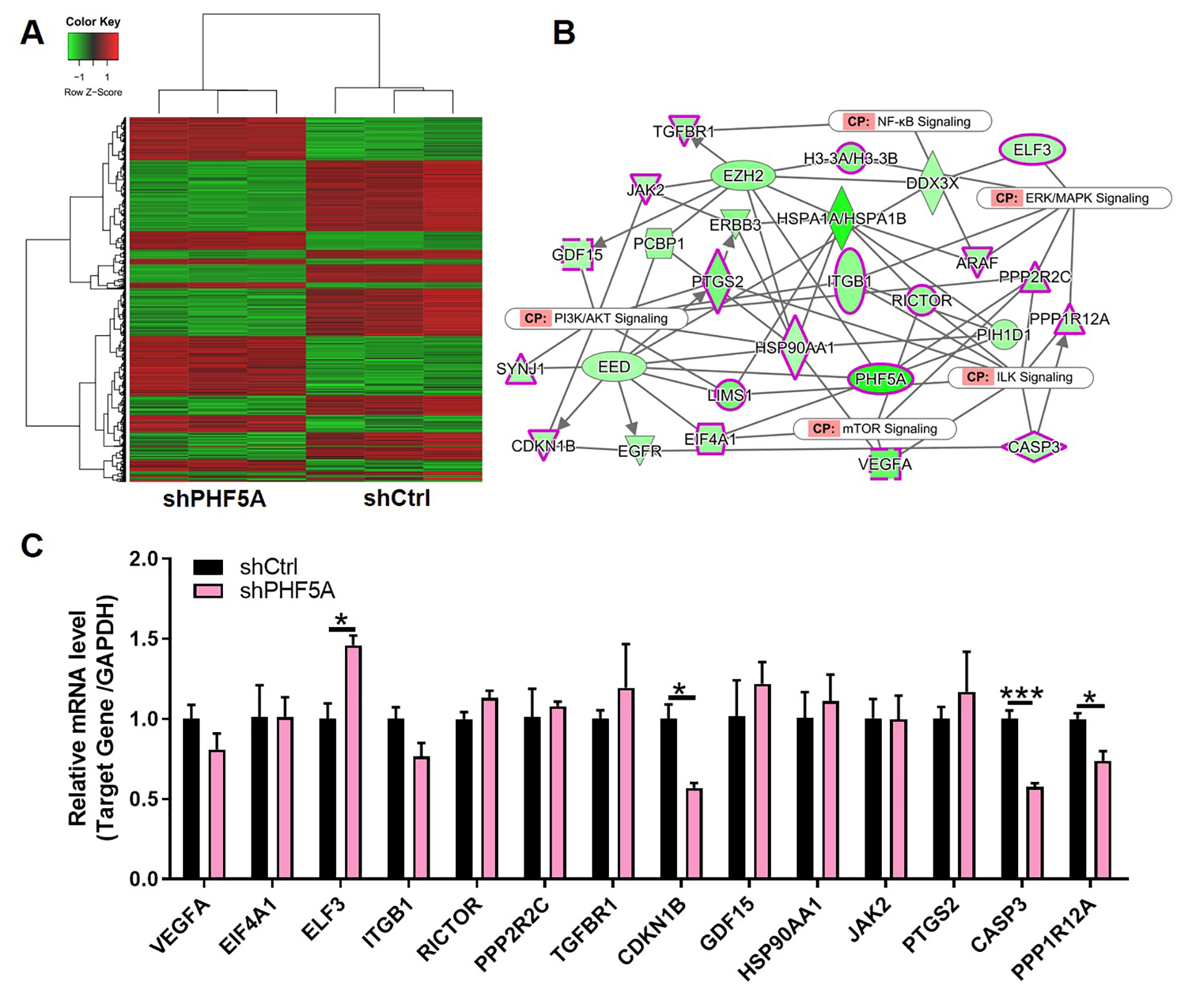


**Fig. S1 Screening of PHF5A downstream targets.** (A) A PrimeView Human Gene Expression Array was performed to identity the differentially expressed genes (DEGs) between shPHF5A and shCtrl groups of Eca-109 cells. (B) Interaction of PHF5A with NF-κB Signaling, ERK/MAPK signaling, PI3K/AKT signaling, ILK Signaling and mTOR Signaling was identified by Ingenuity Pathway Analysis (IPA). (C) Relative mRNA expression levels of several potential targets were validated by qPCR analysis. GAPDH served as the inner control. *p<0.05, ***p<0.001.


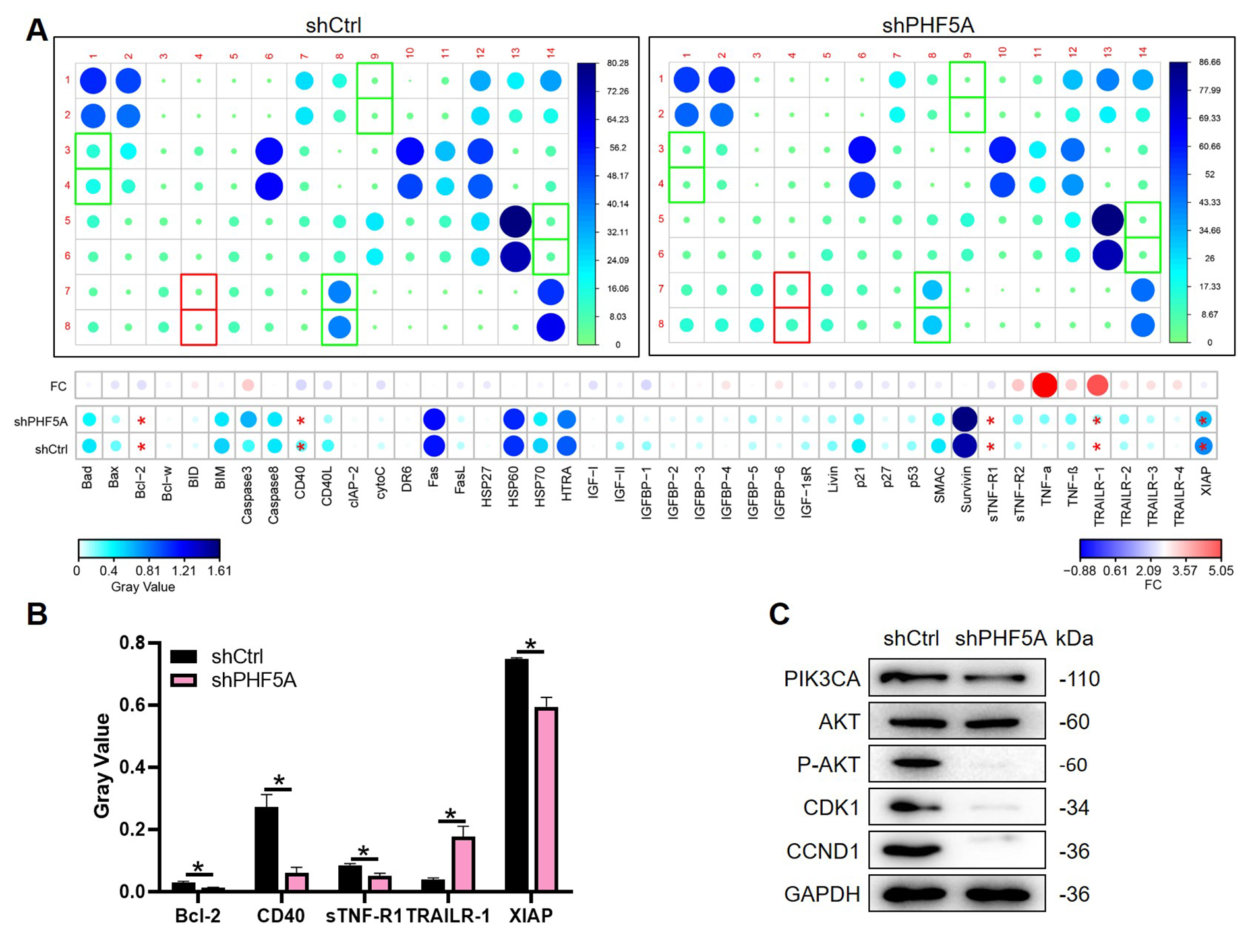


**Fig. S2 Potential regulatory proteins for cell apoptosis.** (A) Human apoptosis antibody assay was used for analyzing apoptosis-related protein level upon PHF5A deficits. (B) Gray values showed significant changes of 5 apoptosis-related proteins. (C) The PI3K/AKT related proteins expression were determined by WB assays. *p<0.05.

**Table S1** Antibodies used in IHC, WB and Co-IP

| Primary antibodies | Dilution in WB | Source species | Company | Catalog No. | Use |
| --- | --- | --- | --- | --- | --- |
| PHF5A | 1:100 | Rabbit | Abcam | Ab193115 | IHC |
| Ki67 | 1:300 | Rabbit | Abcam | Ab16667 | IHC |
| PHF5A | 1:1000 | Rabbit | Abcam | ab116014 | WB |
| Caspase-3 | 1:1000 | Rabbit | CST | 9662S | WB |
| CDKN1B | 1:500 | Rabbit | BOSTER | BM4229 | WB |
| ITGB1 | 1:750 | Mouse | Abcam | Ab8991 | WB |
| VEGFA | 1:1000 | Rabbit | Abcam | ab46154 | WB |
| DYKDDDDK Tag | 1:1000 | Rabbit | CST | 14793 | Co-IP |
| PHF5A | 1:50 | Mouse | Proteintech | 15554-1-AP | Co-IP |
| VEGFA | 1:1000 | Rabbit | Absin | Abs136982 | Co-IP |
| GAPDH | 1:3000 | Mouse | Proteintech | 60004-1-lg | Co-IP |
| MDM2 | 1:3000 | Rabbit | Proteintech | 27883-1-AP | Co-IP |
| Ubiquitin | 1:2000 | Mouse | CST | 3936S | WB |
| AKT | 1:2000 | Rabbit | Abcam | 10176-2-AP | WB |
| P-AKT | 1:2000 | Mouse | Proteintech | 66444-1-Ig | WB |
| mTOR | 1:3000 | Mouse | Proteintech | 66888-1-Ig | WB |
| p-mTOR | 1:1000 | Rabbit | CST | 5536 | WB |
| CDK1 | 1:3000 | Rabbit | Abcam | ab133327 | WB |
| CCND1 | 1:2000 | Rabbit | Abcam | ab134175 | WB |
| PIK3CA | 1:1000 | Rabbit | Abcam | ab40776 | WB |
| Primary antibodies | Dilution in IHC | Source species | Company | Catalog No. |  |
| PHF5A | 1:100 | Rabbit | Abcam | Ab193115 |  |
| Ki67 | 1:300 | Rabbit | Abcam | Ab16667 |  |
| Secondary antibody | Dilution |  | Company | Catalog No. |  |
| HRP Goat Anti-Rabbit IgG (WB) | 1:3000 |  | Beyotime | A0208 |  |
| HRP Goat Anti-Mouse IgG (WB) | 1:3000 |  | Beyotime | A0216 |  |
| HRP Goat Anti-Rabbit IgG (IHC) | 1:400 |  | Abcam | Ab97080 |  |

**Table S2** Target sequences and shRNA sequences

| Gene | | No. | Target sequence (5'-3') | shRNA sequences (5'-3') |
| --- | --- | --- | --- | --- |
| PHF5A | PHF5A-1 | | GCGCATATGTGATGAGTGTAA | ccggGCGCATATGTGATGAGTGTAActcgagTTACACTCATCACATATGCGCtttttg |
| PHF5A | PHF5A-2 | | TAAGACAGACCTCTTCTATGA | ccggTAAGACAGACCTCTTCTATGActcgagTCATAGAAGAGGTCTGTCTTAtttttg |
| PHF5A | PHF5A-3 | | AATGTGATGGCAAGTGTGTGA | ccggAATGTGATGGCAAGTGTGTGActcgagTCACACACTTGCCATCACATTtttttg |
| VEGFA | VEGFA-1 | | CACAACAAATGTGAATGCAGA | ccggCACAACAAATGTGAATGCAGActcgagTCTGCATTCACATTTGTTGTGtttttg |
| VEGFA | VEGFA-2 | | CGAGTACATCTTCAAGCCAT | ccggCGAGTACATCTTCAAGCCATctcgagATGGCTTGAAGATGTACTCGAtttttg |
| VEGFA | VEGFA-3 | | CACCATGCAGATTATGCGGAT | ccggCACCATGCAGATTATGCGGATctcgagATCCGCATAATCTGCATGGTGtttttg |

**Table S3** Primers used in qPCR

| Gene | Forward primer sequence (5’-3’) | Reverse primer sequence (5’-3’) |
| --- | --- | --- |
| PHF5A | ATCTTTTGCCGCAAGCAG | AGAGTGCAGGGACGCACATA |
| VEGFA | CTTGCCTTGCTGCTCTACCT | TTCGTGATGATTCTGCCCTC |
| GAPDH | TGACTTCAACAGCGACACCCA | CACCCTGTTGCTGTAGCCAAA |
| EIF4A1 | TGGATGAAGCTGACGAAATG | CTGGGTGTTGCTGTTGAG |
| ELF3 | GAGAAGAACAAGTACGACGCAAGC | CAGCTCCTCAAGGGCACAATT |
| ITGB1 | CAGTGAATGGGAACAACGAG | TAATGCAAGGCCAATAAGAAC |
| RICTOR | ACGATTTCCTCTGAAGCTCTTG | AAGGTGGGATAACGCTGGTT |
| PPP2R2C | ACAGCACTTTCCAGAGCCACG | GAGCCACTTGATCTTGTTGATCTTCT |
| TGFBR1 | GTCATCACCTGGCCTTGGTC | GGTCCTCTTCATTTGGCACTC |
| CDKN1B | AGGAATAAGGAAGCGACCTGC | TGGGGAACCGTCTGAAACAT |
| GDF15 | GCAAGAACTCAGGACGGTGA | TGGAGTCTTCGGAGTGCAAC |
| HSP90AA1 | TTGTAGACTGCCGAGTAATAGCC | TCCTCATCGCTGCCACTAA |
| JAK2 | CAAACCAAGAGGGTTCAAATG | GCTGGAGGTGCTACTTCTTTAC |
| PTGS2 | CAAATCCTTGCTGTTCCCACC | TTTCTCCATAGAATCCTGTCCG |
| CASP3 | GAATGACATCTCGGTCTGGT | ACATCACGCATCAATTCCAC |
| PPP1R12A | ATCTGCCCTGTAGAGCCTTG | GGGGAAGGAAGGTTGTCCTG |

**Table S4** Pearson correlation analysis between PHF5A expression and tumor characteristics in patients with [esophagus cancer](http://dict.youdao.com/w/glioma/#keyfrom=E2Ctranslation)

| Tumor characteristics | Index | PHF5A |
| --- | --- | --- |
| Tumor [Infiltrate](D:/360%E5%AE%89%E5%85%A8%E6%B5%8F%E8%A7%88%E5%99%A8%E4%B8%8B%E8%BD%BD/Dict/8.4.0.0/resultui/html/index.html#/javascript:;) | Pearson correlation | 0.255 |
|  | Significance (two-tailed) | <0.05 |
|  | N | 69 |
